# A novel algorithm for better distinction of primary mucinous ovarian carcinomas and mucinous carcinomas metastatic to the ovary

**DOI:** 10.1007/s00428-018-2504-0

**Published:** 2019-01-10

**Authors:** Michiel Simons, Thomas Bolhuis, Anton F. De Haan, Annette H. Bruggink, Johan Bulten, Leon F. Massuger, Iris D. Nagtegaal

**Affiliations:** 1 Department of Pathology, Radboud University Medical Center, Nijmegen, 6525 GA The Netherlands; 2 Department for Health Evidence, Radboud University Medical Center, Nijmegen, 6525 GA The Netherlands; 3 PALGA, The Nationwide Network and Registry of Histo- and Cytopathology in the Netherlands, 3995 GA Houten, The Netherlands; 4 Department of Obstetrics and Gynecology, Radboud University Medical Center, Nijmegen, 6525 GA The Netherlands

**Keywords:** Mucinous ovarian carcinoma, Colorectal carcinoma, Metastasis, Algorithm

## Abstract

Primary mucinous ovarian carcinomas (MOC) are notoriously difficult to distinguish from mucinous carcinomas metastatic to the ovary (mMC). Studies performed on small cohorts reported algorithms based on tumor size and laterality to aid in distinguishing MOC from mMC. We evaluated and improved these by performing a large-scale, nationwide search in the Dutch Pathology Registry. All registered pathology reports fulfilling our search criteria concerning MOC in the Netherlands from 2000 to 2011 were collected. Age, histology, laterality, and size were extracted. An existing database covering the same timeline containing tumors metastatic to the ovary was used, extracting all mMC, age, size, laterality, and primary tumor location. Existing algorithms were applied to our cohort. Subsequently, an algorithm based on tumor histology, laterality, and a nomogram based on age and size was created for differentiating MOC and mMC. We identified 735 MOC and 1018 mMC. Patients with MOC were significantly younger and MOC were significantly larger and more often unilateral than mMC. Signet ring cell carcinomas were rarely primary. Our algorithm used signet ring cell histology, bilaterality, and a nomogram integrating patient age and tumor size to diagnose mMC. Sensitivity and specificity for mMC was 90.1% and 59.0%, respectively. Applying existing algorithms on our cohort yielded a far lower sensitivity. The algorithm described here using tumor histology, laterality, size, and patient age has higher sensitivity but lower specificity compared to earlier algorithms and aids in indicating tumor origin, but for conclusive diagnosis, careful integration of morphology, immunohistochemistry, and clinical and imaging data is recommended.

**Electronic supplementary material:**

The online version of this article (10.1007/s00428-018-2504-0) contains supplementary material, which is available to authorized users.

## Introduction

It is well known that a considerable part of mucinous ovarian carcinomas are in fact metastases, mainly from the gastrointestinal tract, pancreas, and gallbladder [1–4]. The distinction of primary mucinous carcinomas of the ovary (MOC) and mucinous carcinomas metastatic to the ovary (mMC) might be difficult and misdiagnosis has important consequences for therapy. Chemotherapy regimens differ between tumor types and advanced stage MOC are generally associated with a poor response to treatment [5]. Although certain histological features may be indicative of primary or metastatic origin, these are often inconclusive [6–9]. A classic immunohistochemical panel of CK7, CK20, and CDx2 is usually considered helpful in indicating tumor origin, but unfortunately shows overlap in expression patterns in MOC and mMC [10–13]. Also, particularly MOC arising from teratomas are known to express a more gastrointestinal phenotype [14].

Macroscopic features as size and laterality have also been investigated. Unilaterality and large size is indicative of MOC, while bilaterality is more suggestive of mMC [7]. We have shown earlier that colorectal mMC are unilateral in almost 60% of cases [1]. Despite this, these macroscopic features have been proposed by various studies as discriminators between MOC and mMC. Seidman et al. proposed an algorithm designating unilateral tumors smaller than 10 cm and bilateral tumors as mMC, and unilateral tumors of at least 10 cm in size as MOC [2]. Another algorithm by Yemelyanova et al. used a different size cut off point of 13 cm [15]. These algorithms classified 90% and 87% of tumors correctly, and yielded a sensitivity for mMC of 94.7% and 82%, respectively. These algorithms should focus on a low rate of false negative patients with mMC, since misdiagnosis will lead to withholding the diagnostic workup to identify a primary tumor elsewhere with important therapeutic and prognostic consequences.

Previous studies were performed on relatively small cohorts. We aimed to evaluate these algorithms on a larger tumor cohort and improve them where possible.

## Materials and methods

### Case selection: primary mucinous ovarian tumors

The nationwide network and registry of histopathology and cytopathology in the Netherlands (PALGA) codes and saves pathology reports in the Netherlands from 1971 with nationwide coverage from 1991 [16]. We performed a nationwide search for primary (micro-)invasive mucinous ovarian carcinomas diagnosed between 2000 and 2011 in the PALGA database obtained by complete resection. All tumors of non-mucinous, mixed, and uncertain histology were excluded. Tumors labeled Krukenberg tumors were excluded from the MOC group, since this term refers to metastatic signet ring cell carcinomas [17]. Tumors associated with pseudomyxoma peritonei (PMP) were excluded. Any tumors of which origin was reported to be uncertain were excluded. History of eligible patients was requested at PALGA, and patients with a history of a gastrointestinal tumor regardless of histology, a mucinous tumor regardless of location, and an adenocarcinoma NOS located in the genital tract were excluded. For each patient, we extracted the following items: age at time of diagnosis, origin (primary or metastasis), histological subtype, laterality, and size of the ovarian tumor. In case of bilateral tumors, both largest and smallest sizes were registered if available. In case of unilateral tumors, size was scored as largest. If only one ovary was resected or reported, we considered the tumor to be unilateral.

### Case selection: mucinous tumors metastatic to the ovary

A database containing mMC was created earlier. Details about criteria for this database are described elsewhere [1]. From this database, we extracted all tumors metastatic to the ovary with histological proof of extra-ovarian origin and mucinous histology. Additional macroscopic data were requested at PALGA. Cases were excluded from this database if tumor size mentioned in additional macroscopy and conclusion was discrepant or if macroscopy contained any information making it uncertain whether a tumor was primary or metastatic. For cases in this dataset, we additionally extracted location of the primary tumor.

These data were combined to create a database containing both MOC and mMC.

### Statistical analysis

For cases with no data available for laterality or size, multiple imputation was applied to estimate these values to maintain cohort size and to avoid biased estimates in the regression analyses. With this technique, multiple complete datasets are created by drawing a value for the missing values based on the estimated distribution. Each dataset is analyzed and the results are combined [18]. Imputed variables and variables used for imputation are shown in Online Resource 1. Twenty imputated datasets were created.

We applied the algorithms described earlier on our database to evaluate sensitivity, specificity, and number of correctly classified cases.

To identify discriminating factors, logistic regression was carried out for each step in the algorithm creation process. Our approach was based on a high sensitivity for mMC.

For creating nomogram scores, regression coefficients B were calculated using logistic regression with the continuous variables age and largest size. For size and age, a score Score_(size + age)_ was calculated for nomogram creation. Details are found in Online Resource 2.

Statistical analysis was performed using IBM SPSS statistics version 20.0. For comparison of means, two-tailed *t* tests were performed, for comparison of frequency distributions between categorical data *χ*^2^ tests were performed. A *p* value < 0.05 was considered statistically significant. ROC curves were used to determine optimal cutoff points.

## Results

### Features of primary mucinous ovarian tumors and tumors metastatic to the ovary

**Fig. 1:**
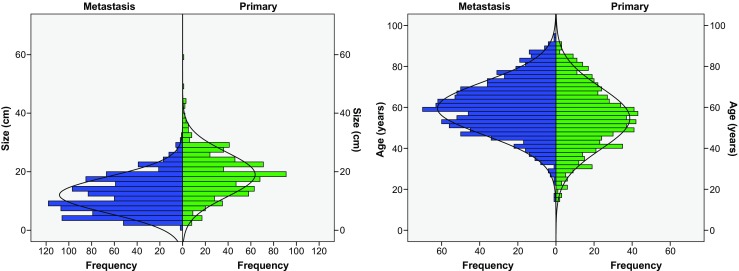
Frequency distribution for largest size (**a**) and age (**b**)

A total of 735 MOC and 1018 mMC were identified. Laterality data was missing for 52 MOC (7.1%) and 84 mMC (8.3%); largest size data were missing for 129 MOC (17.6%) and 312 mMC (30.6%). Patients with MOC were significantly younger than patients with mMC (54.6 vs. 59.6 years; *p* < 0.01) and had larger tumors (19.0 vs. 12.0 cm; *p* < 0.01). Size and age distribution among patients with MOC and mMC are depicted in Fig. 1. Patients with MOC had unilateral tumors in 662 cases (90.1%) vs. 73 (9.9%) bilateral tumors, whereas patients with mMC had bilateral tumors in 508 cases (49.9%) and unilateral in 510 cases (50.1%) (*p* < 0.001). Signet ring cell carcinomas were more often metastatic than primary (122 (98.4%) vs. 2 (1.6%); *p* < 0.001). Bilateral tumors were more often metastatic than primary (508 (87.4%) vs. 73 (12.6%); *p* < 0.001), whereas unilateral tumors were primary in 662 cases (56.5%) and were metastatic in 510 cases (43.5%). Characteristics before and after imputation are shown in Tables 1 and 2, showing that this led to no significant changes.

**Table 1:** Features of primary and metastatic mucinous ovarian carcinomas before imputation, age, and size expressed as mean

Parameter		Primary	%	Metastasis	%	*p* value
Age		54.6 ± 15.1		59.6 ± 13.1		< 0.001
Histology	Mucinous	733	45.0	896	55.0	< 0.001
	Signet-ring cell	2	1.6	122	98.4	
Location primary tumor	Appendix			97	9.5	
	Bladder			2	0.2	
	Breast			3	0.3	
	Cervix			2	0.2	
	Endometrium			4	0.4	
	Colon			748	73.5	
	Duodenum			1	0.1	
	Small intestine			22	2.2	
	Pancreas			17	1.7	
	Bile ducts/gallbladder			14	1.4	
	Esophagus			7	0.7	
	Stomach			100	9.8	
	Urachus			1	0.1	
Laterality	Left	284	56.6	218	43.4	< 0.001
	Right	330	54.1	280	45.9	
	Bilateral	69	13.7	436	86.3	
	Unknown	52	38.2	84	61.7	
Size (largest)		18.9 ± 7.9		11.6 ± 6.4		< 0.001
Total		735	41.9	1018	58.1

**Table 2:** Size and laterality of primary and metastatic mucinous ovarian carcinomas after imputation

Parameter		Primary	%	Metastasis	%	*p* value
Laterality	Left	307	57.8	224	42.2	< 0.001
	Right	355	55.4	286	44.6	
	Bilateral	73	12.6	508	87.4	
	Unknown	0		0		
Size (largest)		19.0		12.0		< 0.001
Total		735	41.9	1018	58.1	

### Comparison to earlier studies

Seidman et al. [2] classified tumors as MOC if they were unilateral and ≥ 10 cm. In our cohort, 15.4% of tumors < 10 cm were primary and 84.6% was metastatic. Tumors ≥ 10 cm were primary in 52.5% and were metastatic in 47.5%. MMC were < 10 cm in 41.5% and ≥ 10 cm in 58.5%. Of MOC, this was 10.5% and 89.5%, respectively. On our data, the Seidman algorithm has a sensitivity of 72.5% and a specificity of 82.4% and of all 76.6% tumors were classified correctly.

Yemelyanova et al. [15] used 13 cm as a size cutoff point. In our cohort, tumors < 13 cm were primary in 26.8% and were metastatic in 73.2%. Tumors ≥ 13 cm were primary in 56.9% and were metastatic in 43.1%. MMC were < 13 cm in 56.8% and ≥ 13 cm in 43.2%. Of MOC, this was 21.1% and 78.9%, respectively. On our data, the Yemelyanova algorithm has a sensitivity of 79.9% and a specificity of 73.6% and of all tumors 77.2% were classified correctly.

Further test details for both algorithms are shown in Table 3.

**Table 3:** Results of algorithms on current tumor cohort

Study		Origin	
		Primary	Metastasis
Seidman et al.	Primary	604	280
	Metastasis	131	738
	Sensitivity	72.4%	
	Specificity	82.2%	
Yemelyanova et al.	Primary	541	205
	Metastasis	194	813
	Sensitivity	79.9%	
	Specificity	73.6%	
Current study	Primary	434	101
	Metastasis	301	917
	Sensitivity	90.1%	
	Specificity	59.0%	

### Optimizing algorithm

Logistic regression identified age, largest size, histology, and laterality as significant independent predicting factors for distinguishing MOC from mMC. Regression coefficients, odds ratios, and 95% confidence intervals are displayed in Online Resource 2.

Signet ring cell histology compared to non-signet ring cell histology showed a sensitivity of only 12.0%, but a specificity of 99.7% for indicating metastasis, with a positive predictive value for metastasis of 98.4%. Comparing bilaterality to unilaterality as a next step, after excluding signet ring cell carcinomas, shows a sensitivity of only 48.1%, but a specificity of 90.0% for indicating metastasis, with a positive predictive value of 85.5%.

Based on the remaining cases, areas under the curve (AUC) for largest size and age as a determinant of origin were 0.78 and 0.64, respectively. To test a combination of these two variables, logistic regression including age and largest size was carried out and rendered regression coefficient B_size_ 0.154 and B_age_ − 0.033, respectively (*p* < 0.001 for both variables). Larger tumors and lower age tended to be associated with primary tumors, although distributions showed too much overlap to be used as a solitary determinant (see Fig. 1). The largest size range was 1 to 60 cm and age range was 15 to 95 years. Exact calculations can be found in Online Resource 3. Final scores for size and age can be found in Online Resources 4 and 5, respectively.

**Fig. 2:**
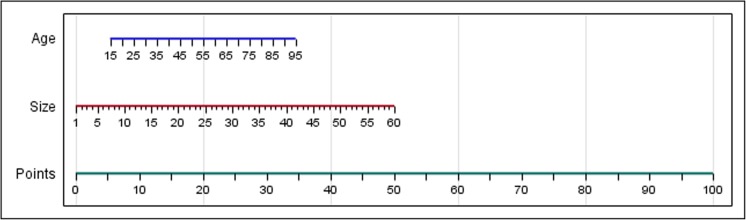
Nomogram based on Score_(size + age)_. By applying patient age en tumor size to the corresponding axes and extrapolating a line through these points to the lower axis, final Score_(size + age)_ can be determined

**Fig. 3:**
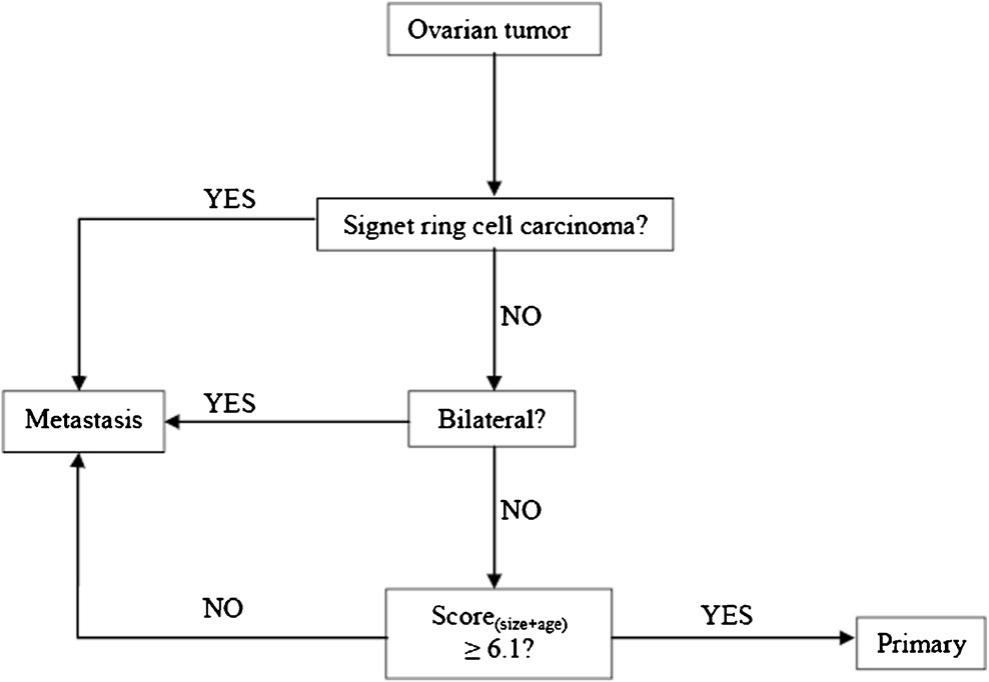
Final algorithm for distinguishing primary mucinous carcinomas and carcinomas metastatic to the ovary using parameters signet ring cells, laterality, patient age, and tumor size. For calculating Score_(size + age)_, use the nomogram displayed in Fig. 2

The ROC curve for Score_(size + age)_ showed an AUC of 0.81 (see Online Resource 6), and for Score_(size)_ or Score_(age)_ again 0.78 and 0.64, respectively. Based on the AUC, Score_(size + age)_ was considered superior to Score_(size)_ or Score_(age)_ separately. An optimal cutoff point for the sum of these scores was determined as 6.1 using the ROC curve coordinates. A nomogram based on this score is shown in Fig. 2. The final algorithm as depicted in Fig. 3 shows a sensitivity and specificity of 90.1% and 59.0%, respectively, and 77.1% of tumors were classified correctly. Details are shown in Table 3.

## Discussion

MOC are often difficult to distinguish from mMC, since morphological and immunohistochemical features are unsatisfactory differentiators. In the current study, we composed the largest database of MOC and mMC to our knowledge to evaluate size and laterality as predictors of tumor origin. Patients with MOC were significantly younger, and MOCs were larger and more often unilateral, which is in line with earlier findings [7, 8]. We compared our data to earlier algorithms using these features and optimized the algorithm by adding presence of signet ring cells and patient age.

Earlier algorithms, based on small patient cohorts, of only 50, 194, and 68 tumors, respectively, solely used laterality and size of the tumors [2, 15, 19]. Application of a 10-cm cutoff in two studies resulted in a sensitivity of 83–95% [2, 19]; adjustment of the cutoff to 13 cm showed a 82% sensitivity [15]. The populations used in these studies were heterogeneous because of diverse inclusion criteria regarding tumors of uncertain primary site and endometrioid and signet ring cell histology. Signet ring cell carcinoma can be of primary ovarian origin, but this is extremely rare [20]. In our cohort, less than 2 per 100 signet ring cell carcinomas were MOC. Applying the earlier algorithms to our cohort yielded far lower sensitivity compared to our algorithm, suggesting that our algorithm including signet ring cells and a combination of relative values for tumor size and patient age renders superior results. Interestingly, sensitivity was also lower than found in the cohorts used in their original studies. Since the number of correctly classified tumors in general was comparable (approximately 77%), these differences seem to be mainly the consequence of different composition of the cohorts. This can be explained by several factors. Firstly, the distribution of primary tumors in the mMC group differs between study populations, which may be due to geographical differences. Secondly, revision of cases in our cohort is not feasible due to large numbers, but since it concerns a nation-wide population-based cohort, it reflects daily practice. Thirdly, patients from tertiary referral centers include a selection of patients, with unusual cases, as can be observed in the Yemelyanova study, that included as much as 35% consultation cases.

No bilateral MOC were observed in the Yemelyanova cohort, as opposed to both our cohort (8.9% bilateral MOC) and the cohorts of Seidman and Khunarmonpong (17% and 12.5%, respectively) [2, 19]. Bilaterality of MOC might be explained by MOC metastasizing from one ovary to the contralateral ovary without this being recognized or reported as such. The possibility of a misdiagnosed mMC cannot be fully excluded. In the current study, the number of bilateral mMC was much lower with 49.9%, most likely due to the large number of colorectal metastases in our cohort which are known for their ability to present as large, unilateral metastases [21]. Another difference is that Yemelyanova et al. also included atypical proliferative mucinous (borderline) tumors (APMTs) and tumors associated with PMP. The latter may have led to a higher number of bilateral metastatic tumors, since we discarded cases associated with PMP. Ovarian involvement of pseudomyxoma peritonei has been shown to be almost invariably of appendiceal origin, the only potential but rare exception being a mucinous neoplasm originating in an ovarian teratoma [14, 22–24]. Hence, cases associated with pseudomyxoma peritonei pose less diagnostic problems. Also, pathological classification of pseudomyxoma peritonei remains problematic [25–27]. We also excluded APMTs to prevent contamination of the MOC group with misclassified mMC, since APMTs would not generally trigger workup for metastasis from a primary tumor elsewhere. Especially pancreatic tumors are known for their capability to mimic APMTs of the ovary. In addition, we ideally wanted to include carcinomas according to WHO criteria, but for micro-invasion varying criteria are used and the exact proportions of invasive foci were rarely reported. To prevent exclusion of actual invasive carcinomas falsely diagnosed as micro-invasive, we did include tumors reported to be micro-invasive.

In our cohort, size and patient age were significantly different between MOC an mMC, but showed too much overlap to be discriminating by themselves. We integrated age in the existing algorithm, using it in direct combination with size. The optimized algorithm based on our own cohort led to a sensitivity and specificity of 90.1% and 59.0%, respectively. Since misdiagnosing an mMC as an MOC has greater consequences for further diagnostic workup and therapy than vice versa, our approach was based on a high sensitivity for diagnosing mMC and yielding a low number of false negative patients. This reduces the possibility of patients ultimately receiving inappropriate treatment for their disease, which differs considerably. Primary mucinous ovarian carcinomas are primarily treated surgically, followed by a combination of paclitaxel- and platinum-based chemotherapy in case of advanced stage disease. In case of mMC, patients will be surgically treated if possible, followed by up to triple therapy with platinum-based chemotherapy, fluoropyrimidines, irinotecan, and the addition of targeted therapy if indicated. With a positive predictive value of 75.3% for mMC, almost 25% of patients will undergo unnecessary diagnostic workup. In the intraoperative setting the algorithm has limited value, since low specificity might lead to denial of surgical staging of patients with MOC when a mMC is reported. However, in practice, manual exploration of the abdominal cavity is performed, which—given the high incidence of both colorectal and appendiceal metastasis—can lead to clinical confirmation of metastasis. In absence of this clinical confirmation, limited staging can be performed, and the surgeon can consider to perform (limited) surgical staging based on the intraoperative suspicion.

In this study, we used multiple imputations to replace values missing at random (MAR) by values drawn from an estimated distribution of the variable in question, a method used frequently in biomedical research [28–30]. This technique is based on the general statistical principle that every subject in a randomly chosen sample can be replaced by a new subject that is randomly chosen from the same source population. Analysis of available cases when values are MAR is no longer based on a random sample from the source population, leading to severely biased study associations and incorrect standard errors. This can be reliably overcome by multiple imputations, rendering this method superior to complete cases analysis [31, 32]. The imprecision caused by the fact that the distribution of the variables with missing values is estimated, is taken into account by creating multiple imputated datasets and combining these to obtain a pooled estimate of the parameters and standard errors [32]. The random subset of which new subjects are chosen or imputed is defined by the already known characteristics (the variables used for imputation). Using as many as six variables for imputation greatly reduces the influence of the technique on the final result [33]. Our algorithm was not subjected to validation, since there is a lack of large validation sets for this type of patient cohort. This might lead to overestimated accuracy, although due to the large sample size this overestimation will be relatively small.

Lack of a gold standard for classifying MOC and mMC causes difficulties in creating study populations in all reported studies to date. We did not revise the cases included in our cohort. We used a proven primary tumor elsewhere as evidence for metastatic ovarian disease, which can be considered an objective criterion. The probability of patients presenting with both a MOC and a gastrointestinal tumor simultaneously seems very low. It is conceivable that metastatic disease may have been falsely classified as a primary ovarian tumor, if no diagnostic workup took place because of initial misdiagnosis or if patients did not undergo surgery of the primary tumor. However, the large sample size reduces the influence of these factors to some extent.

Evaluation of histological and immunohistochemical features as well as clinical and imaging data was impeded by the large sample size and therefore considered beyond the scope of this study. Microscopic features observed more often in MOC are for example expansive growth patterns or presence of precursor lesions, whereas features such as infiltrative growth, dirty necrosis, lymph vessel invasion, and surface involvement are seen more often in mMC [7–9]. Multiple studies have shown that MOC and mMC show overlap in classic immunohistochemical expression patterns [10–13]. Despite overlap of these morphological and immunophenotypical features between MOC and mMC, integrating histological and immunohistochemical features will very probably further optimize the described algorithm. Also, recently discovered markers may prove superior to the existing combinations, such as SATB2 which is a promising new marker with high specificity for gastrointestinal origin [34–36]. This algorithm can be useful for frozen section, although strictly for patients with unilateral salpingo-oophorectomy it may be misleading since microscopic involvement of the contralateral ovary may not be macroscopically visible preoperatively and therefore prevent bilateral resection.

In conclusion, our algorithm has a higher sensitivity of 90.1% for diagnosing mMC compared to earlier reported algorithms, hereby validating these earlier approaches on a large cohort and adding patient age and tumor histology as contributing factors. Macroscopic and demographic features as proposed in the current study strongly aid in decision making, but algorithms as described here should be regarded as helpful rather than conclusive tools. Ultimately, differentiating MOC from mMC is a task beyond the responsibility of the pathologist alone and should be based on careful integration of preoperative workup including imaging and laboratory results and macroscopic, histological, and immunophenotypical tumor features and requires accurate and thorough multidisciplinary communication.

## Electronic supplementary material

Online Resource 1 Imputed variables and variables used for imputation (PDF 5.90 kb)

Online Resource 2 Regression coefficients, odds ratios and 95% confidence intervals of multivariate regression analysis in all phases of algorithm development. All values were statistically significant (*p* < 0.001). (PDF 10 kb)

Online Resource 3 Formulas and calculations for nomogram scores (PDF 114 kb)

Online Resource 4 Nomogram scores for size (PDF 28.2 kb)

Online Resource 5 Nomogram scores for age (PDF 37.3 kb)

Online Resource 6 ROC curve for score_(size + age) (PDF 29.2 kb)_

## References

[1] Bruls J, Simons M, Overbeek LI, Bulten J, Massuger LF, Nagtegaal ID (2015). A national population-based study provides insight in the origin of malignancies metastatic to the ovary. Virchows Arch.

[2] Seidman JD, Kurman RJ, Ronnett BM (2003). Primary and metastatic mucinous adenocarcinomas in the ovaries: incidence in routine practice with a new approach to improve intraoperative diagnosis. Am J Surg Pathol.

[3] Moore RG, Chung M, Granai CO, Gajewski W, Steinhoff MM (2004). Incidence of metastasis to the ovaries from nongenital tract primary tumors. Gynecol Oncol.

[4] Antila R, Jalkanen J, Heikinheimo O (2006). Comparison of secondary and primary ovarian malignancies reveals differences in their pre- and perioperative characteristics. Gynecol Oncol.

[5] Simons M, Ezendam N, Bulten J, Nagtegaal I, Massuger L (2015). Survival of patients with mucinous ovarian carcinoma and ovarian metastases: a population-based Cancer registry study. Int J Gynecol Cancer.

[6] McCluggage WG, Wilkinson N (2005). Metastatic neoplasms involving the ovary: a review with an emphasis on morphological and immunohistochemical features. Histopathology.

[7] Lee KR, Young RH (2003). The distinction between primary and metastatic mucinous carcinomas of the ovary: gross and histologic findings in 50 cases. Am J Surg Pathol.

[8] Riopel MA, Ronnett BM, Kurman RJ (1999). Evaluation of diagnostic criteria and behavior of ovarian intestinal-type mucinous tumors: atypical proliferative (borderline) tumors and intraepithelial, microinvasive, invasive, and metastatic carcinomas. Am J Surg Pathol.

[9] Lewis MR, Deavers MT, Silva EG, Malpica A (2006). Ovarian involvement by metastatic colorectal adenocarcinoma: still a diagnostic challenge. Am J Surg Pathol.

[10] Vang R, Gown AM, Wu LS, Barry TS, Wheeler DT, Yemelyanova A, Seidman JD, Ronnett BM (2006). Immunohistochemical expression of CDX2 in primary ovarian mucinous tumors and metastatic mucinous carcinomas involving the ovary: comparison with CK20 and correlation with coordinate expression of CK7. Mod Pathol.

[11] Vang R, Gown AM, Barry TS, Wheeler DT, Yemelyanova A, Seidman JD, Ronnett BM (2006). Cytokeratins 7 and 20 in primary and secondary mucinous tumors of the ovary: analysis of coordinate immunohistochemical expression profiles and staining distribution in 179 cases. Am J Surg Pathol.

[12] Groisman GM, Meir A, Sabo E (2004). The value of Cdx2 immunostaining in differentiating primary ovarian carcinomas from colonic carcinomas metastatic to the ovaries. Int J Gynecol Pathol.

[13] Werling RW, Yaziji H, Bacchi CE, Gown AM (2003). CDX2, a highly sensitive and specific marker of adenocarcinomas of intestinal origin: an immunohistochemical survey of 476 primary and metastatic carcinomas. Am J Surg Pathol.

[14] Vang R, Gown AM, Zhao C, Barry TS, Isacson C, Richardson MS, Ronnett BM (2007). Ovarian mucinous tumors associated with mature cystic teratomas: morphologic and immunohistochemical analysis identifies a subset of potential teratomatous origin that shares features of lower gastrointestinal tract mucinous tumors more commonly encountered as secondary tumors in the ovary. Am J Surg Pathol.

[15] Yemelyanova AV, Vang R, Judson K, Wu LS, Ronnett BM (2008). Distinction of primary and metastatic mucinous tumors involving the ovary: analysis of size and laterality data by primary site with reevaluation of an algorithm for tumor classification. Am J Surg Pathol.

[16] Casparie M, Tiebosch AT, Burger G, Blauwgeers H, van de Pol A, van Krieken JH, Meijer GA (2007). Pathology databanking and biobanking in the Netherlands, a central role for PALGA, the nationwide histopathology and cytopathology data network and archive. Cell Oncol.

[17] Vang RCA, Kommoss F, Matias-Guiu X, Ronnett BM, Young RH, Kurman RJCM, Herrington CS, Young RH (2014). Secondary Tumours. WHO classification of Tumours of female reproductive organs.

[18] van Buuren S (2007). Multiple imputation of discrete and continuous data by fully conditional specification. Stat Methods Med Res.

[19] Khunamornpong S, Suprasert P, Pojchamarnwiputh S, Na Chiangmai W, Settakorn J, Siriaunkgul S (2006). Primary and metastatic mucinous adenocarcinomas of the ovary: evaluation of the diagnostic approach using tumor size and laterality. Gynecol Oncol.

[20] McCluggage WG, Young RH (2008). Primary ovarian mucinous tumors with signet ring cells: report of 3 cases with discussion of so-called primary Krukenberg tumor. Am J Surg Pathol.

[21] Judson K, McCormick C, Vang R, Yemelyanova AV, Wu LS, Bristow RE, Ronnett BM (2008). Women with undiagnosed colorectal adenocarcinomas presenting with ovarian metastases: clinicopathologic features and comparison with women having known colorectal adenocarcinomas and ovarian involvement. Int J Gynecol Pathol.

[22] Ronnett BM, Shmookler BM, Diener-West M, Sugarbaker PH, Kurman RJ (1997). Immunohistochemical evidence supporting the appendiceal origin of pseudomyxoma peritonei in women. Int J Gynecol Pathol.

[23] Young RH, Gilks CB, Scully RE (1991). Mucinous tumors of the appendix associated with mucinous tumors of the ovary and pseudomyxoma peritonei. A clinicopathological analysis of 22 cases supporting an origin in the appendix. Am J Surg Pathol.

[24] Ronnett BM, Seidman JD (2003). Mucinous tumors arising in ovarian mature cystic teratomas: relationship to the clinical syndrome of pseudomyxoma peritonei. Am J Surg Pathol.

[25] Bradley RF, Stewart JH, Russell GB, Levine EA, Geisinger KR (2006). Pseudomyxoma peritonei of appendiceal origin: a clinicopathologic analysis of 101 patients uniformly treated at a single institution, with literature review. Am J Surg Pathol.

[26] Ronnett BM (2006). Pseudomyxoma peritonei: a rose by any other name. Am J Surg Pathol.

[27] Shetty S, Natarajan B, Thomas P, Govindarajan V, Sharma P, Loggie B (2013). Proposed classification of pseudomyxoma peritonei: influence of signet ring cells on survival. Am Surg.

[28] Allard MA, Adam R, Giuliante F, Lapointe R, Hubert C, Ijzermans JNM, Mirza DF, Elias D, Laurent C, Gruenberger T, Poston G, Letoublon C, Isoniemi H, Lucidi V, Popescu I, Figueras J (2017). Long-term outcomes of patients with 10 or more colorectal liver metastases. Br J Cancer.

[29] Lorincz AT, Nathan M, Reuter C, Warman R, Thaha MA, Sheaff M, Vasiljevic N, Ahmad A, Cuzick J, Sasieni P (2017). Methylation of HPV and a tumor suppressor gene reveals anal cancer and precursor lesions. Oncotarget.

[30] Jansen IGH, Mulder M, Goldhoorn RB, investigators MCR (2018). Endovascular treatment for acute ischaemic stroke in routine clinical practice: prospective, observational cohort study (MR CLEAN registry). BMJ.

[31] Janssen KJ, Donders AR, Harrell FE, Vergouwe Y, Chen Q, Grobbee DE, Moons KG (2010). Missing covariate data in medical research: to impute is better than to ignore. J Clin Epidemiol.

[32] Donders AR, van der Heijden GJ, Stijnen T, Moons KG (2006). Review: a gentle introduction to imputation of missing values. J Clin Epidemiol.

[33] Lang KM, Little TD (2018). Principled missing data treatments. Prev Sci.

[34] Moh M, Krings G, Ates D, Aysal A, Kim GE, Rabban JT (2016). SATB2 expression distinguishes ovarian metastases of colorectal and Appendiceal origin from primary ovarian tumors of mucinous or Endometrioid type. Am J Surg Pathol.

[35] Strickland S, Wasserman JK, Giassi A, Djordjevic B, Parra-Herran C (2016). Immunohistochemistry in the diagnosis of mucinous neoplasms involving the ovary: the added value of SATB2 and biomarker discovery through protein expression database mining. Int J Gynecol Pathol.

[36] Perez Montiel D, Arispe Angulo K, Cantu-de Leon D, Bornstein Quevedo L, Chanona Vilchis J, Herrera Montalvo L (2015). The value of SATB2 in the differential diagnosis of intestinal-type mucinous tumors of the ovary: primary vs metastatic. Ann Diagn Pathol.

