# Supplementary material for: A novel algorithm for better distinction of primary mucinous ovarian carcinomas and mucinous carcinomas metastatic to the ovary
Source: Virchows Arch. 2019 Jan 10;474(3):289–96. doi: 10.1007/s00428-018-2504-0 (PMC6515884; doi:10.1007/s00428-018-2504-0)
Supplement: Supplementary file 1 — Imputed variables and variables used for imputation (PDF 5.90 kb) [file 428_2018_2504_MOESM1_ESM.pdf]

**Imputated variables**	**Variables used for imputation**
Largest size	Age
Laterality	Origin (metastasis or primary)
	Location of primary tumor
	Histology
	Largest size
	Laterality
