# Supplementary material for: A novel algorithm for better distinction of primary mucinous ovarian carcinomas and mucinous carcinomas metastatic to the ovary
Source: Virchows Arch. 2019 Jan 10;474(3):289–96. doi: 10.1007/s00428-018-2504-0 (PMC6515884; doi:10.1007/s00428-018-2504-0)
Supplement: Supplementary file 2 — Regression coefficients, odds ratios and 95% confidence intervals of multivariate regression analysis in all phases of algorithm development. All values were statistically significant (p < 0.001). (PDF 10 kb) [file 428_2018_2504_MOESM2_ESM.pdf]

All cases	Variable	Regression coefficient (B)	Odds ratio	95% CI
	Subtype	3.524	33.92	8.01-143.70
	Age	-0.024	0.976	0.968-0.985
	Laterality	-1.867	0.155	0.113-0.211
	Largest size	0.129	1.137	1.115-1.160
**After exclusion of signet ring cell histology**	Age	-0.024	0.976	0.968-0.985
	Laterality	-1.858	0.156	0,114-0.213
	Largest size	0.129	1.137	1.115-1.160
**After exclusion of signet ring cell histology and bilaterality**	Age	-0.033	0.968	0.959-0.978
	Largest size	0.154	1.166	1.137-1.192
