# Supplementary material for: A novel algorithm for better distinction of primary mucinous ovarian carcinomas and mucinous carcinomas metastatic to the ovary
Source: Virchows Arch. 2019 Jan 10;474(3):289–96. doi: 10.1007/s00428-018-2504-0 (PMC6515884; doi:10.1007/s00428-018-2504-0)
Supplement: Supplementary file 3 — Formulas and calculations for nomogram scores (PDF 114 kb) [file 428_2018_2504_MOESM3_ESM.pdf]

The following formula was used to calculate a relative score per size value ranging from 0 to 100:

$$Score(size) = 100 * \left(\frac{(Size * Bsize) - S1}{SN - S1}\right)$$

For age a score relative to size was calculated per age value using the formula:

$$Score(age) = 100 * \left(\frac{Age * Bage}{SN - S1}\right)$$

with SN being the largest size and S1 being the smallest size in the cohort multiplied by $B_{size}$. Per patient, these scores were added to yield a total score $Score_{(size+age)}$.

For size, a relative score was calculated per size value ranging from 0 to 100 using the following formula:

$$Score(size) = 100 * \left(\frac{(Size - 1) * 0.154)}{9.086 - 0.154}\right)$$

For age a score relative to size was calculated per age value using the following formula

$$Score(age) = 100 * \left(\frac{Age * -0.033}{9.086 - 0.154}\right)$$

These scores are shown in Supplemental Tables 1 and 2. Per patient, these scores were added to yield a total score $Score_{(size+age)}$.
