# Supplementary material for: A novel algorithm for better distinction of primary mucinous ovarian carcinomas and mucinous carcinomas metastatic to the ovary
Source: Virchows Arch. 2019 Jan 10;474(3):289–96. doi: 10.1007/s00428-018-2504-0 (PMC6515884; doi:10.1007/s00428-018-2504-0)
Supplement: Supplementary file 4 — Nomogram scores for size (PDF 28.2 kb) [file 428_2018_2504_MOESM4_ESM.pdf]

Size (cm)	S(N)	Score$_{(size)}$
1	0,2	0,0
2	0,3	1,7
3	0,5	3,4
4	0,6	5,1
5	0,8	6,8
6	0,9	8,5
7	1,1	10,2
8	1,2	11,9
9	1,4	13,6
10	1,5	15,3
11	1,7	16,9
12	1,8	18,6
13	2,0	20,3
14	2,2	22,0
15	2,3	23,7
16	2,5	25,4
17	2,6	27,1
18	2,8	28,8
19	2,9	30,5
20	3,1	32,2
21	3,2	33,9
22	3,4	35,6
23	3,5	37,3
24	3,7	39,0
25	3,9	40,7
26	4,0	42,4
27	4,2	44,1
28	4,3	45,8
29	4,5	47,5
30	4,6	49,2
31	4,8	50,8
32	4,9	52,5
33	5,1	54,2
34	5,2	55,9
35	5,4	57,6
36	5,5	59,3
37	5,7	61,0
38	5,9	62,7
39	6,0	64,4
40	6,2	66,1
41	6,3	67,8
42	6,5	69,5
43	6,6	71,2
44	6,8	72,9
45	6,9	74,6
46	7,1	76,3
47	7,2	78,0
48	7,4	79,7
49	7,5	81,4
50	7,7	83,1
51	7,9	84,7
52	8,0	86,4
53	8,2	88,1
54	8,3	89,8
55	8,5	91,5
56	8,6	93,2
57	8,8	94,9
58	8,9	96,6
59	9,1	98,3
60	9,2	100,0
