# Supplementary material for: A novel algorithm for better distinction of primary mucinous ovarian carcinomas and mucinous carcinomas metastatic to the ovary
Source: Virchows Arch. 2019 Jan 10;474(3):289–96. doi: 10.1007/s00428-018-2504-0 (PMC6515884; doi:10.1007/s00428-018-2504-0)
Supplement: Supplementary file 5 — Nomogram scores for age (PDF 37.3 kb) [file 428_2018_2504_MOESM5_ESM.pdf]

Age (y)	S(N)	$S_{(age)}$
15	-0,5	-5,4
16	-0,5	-5,8
17	-0,6	-6,2
18	-0,6	-6,5
19	-0,6	-6,9
20	-0,7	-7,3
21	-0,7	-7,6
22	-0,7	-8,0
23	-0,8	-8,4
24	-0,8	-8,7
25	-0,8	-9,1
26	-0,9	-9,4
27	-0,9	-9,8
28	-0,9	-10,2
29	-1,0	-10,5
30	-1,0	-10,9
31	-1,0	-11,3
32	-1,1	-11,6
33	-1,1	-12,0
34	-1,1	-12,3
35	-1,2	-12,7
36	-1,2	-13,1
37	-1,2	-13,4
38	-1,3	-13,8
39	-1,3	-14,2
40	-1,3	-14,5
41	-1,4	-14,9
42	-1,4	-15,3
43	-1,4	-15,6
44	-1,5	-16,0
45	-1,5	-16,3
46	-1,5	-16,7
47	-1,6	-17,1
48	-1,6	-17,4
49	-1,6	-17,8
50	-1,7	-18,2
51	-1,7	-18,5
52	-1,7	-18,9
53	-1,7	-19,2
54	-1,8	-19,6
55	-1,8	-20,0
56	-1,8	-20,3
57	-1,9	-20,7
58	-1,9	-21,1
59	-1,9	-21,4
60	-2,0	-21,8
61	-2,0	-22,2
62	-2,0	-22,5
63	-2,1	-22,9
64	-2,1	-23,2
65	-2,1	-23,6
66	-2,2	-24,0
67	-2,2	-24,3
68	-2,2	-24,7
69	-2,3	-25,1
70	-2,3	-25,4
71	-2,3	-25,8
72	-2,4	-26,2
73	-2,4	-26,5
74	-2,4	-26,9
75	-2,5	-27,2
76	-2,5	-27,6
77	-2,5	-28,0
78	-2,6	-28,3
79	-2,6	-28,7
80	-2,6	-29,1
81	-2,7	-29,4
82	-2,7	-29,8
83	-2,7	-30,1
84	-2,8	-30,5
85	-2,8	-30,9
86	-2,8	-31,2
87	-2,9	-31,6
88	-2,9	-32,0
89	-2,9	-32,3
90	-3,0	-32,7
91	-3,0	-33,1
92	-3,0	-33,4
93	-3,1	-33,8
94	-3,1	-34,1
95	-3,1	-34,5

.
