# Supplementary figures and images for: A novel algorithm for better distinction of primary mucinous ovarian carcinomas and mucinous carcinomas metastatic to the ovary
Source: Virchows Arch. 2019 Jan 10;474(3):289–96. doi: 10.1007/s00428-018-2504-0 (PMC6515884; doi:10.1007/s00428-018-2504-0)

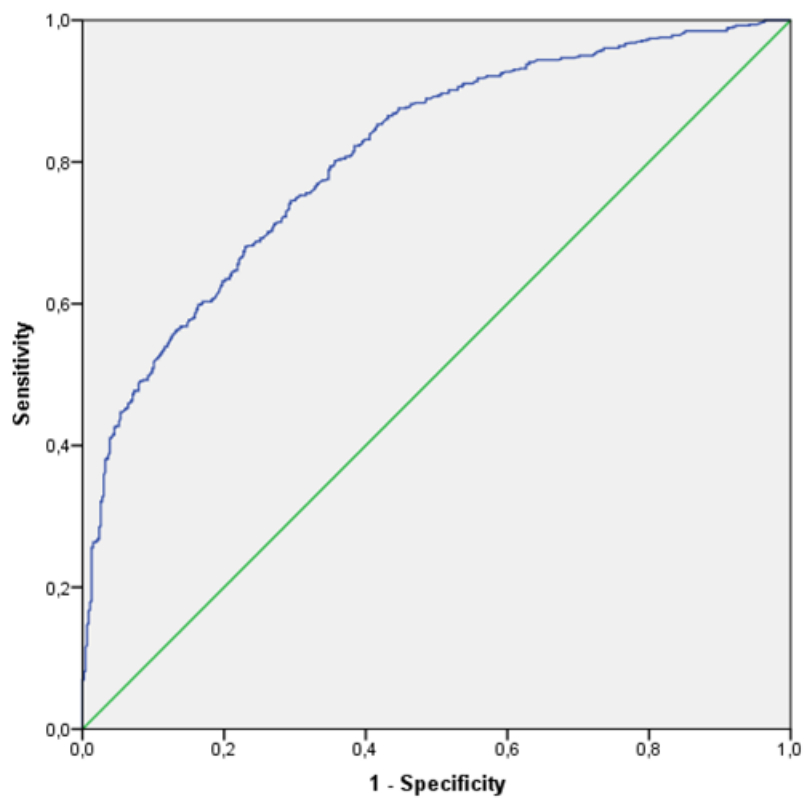

Supplement: Supplementary file 6 — ROC curve for score(size + age) (PDF 29.2 kb) [file 428_2018_2504_MOESM6_ESM.pdf]
